# Supplementary material for: In-House Filtration Efficiency Assessment of Vapor Hydrogen Peroxide Decontaminated Filtering Facepiece Respirators (FFRs)
Source: Int J Environ Res Public Health. 2021 Jul 4;18(13):7169. doi: 10.3390/ijerph18137169 (PMC8297238; doi:10.3390/ijerph18137169)
Supplement: Supplementary file 1 [file ijerph-18-07169-s001.zip › ijerph-1215815-supplementary materials.pdf]

Supplementary Material 1: The 3D digital design of the air duct is in STL format (the file will beis submitted along with the manuscript in a single file).

Supplementary Material 2. Filtration efficiency (FE) comparison between 1 and 10 minutes sampling times for KN95 (n=5) and N95-8210 (n=5) FFRs.

**Particle size (μm)**	**FE of KN95 (standard error)**			**FE of KN95 FFR (standard error)**		
	**Sampling time (minutes)***			**Sampling time (minutes)***		
	**1**	**10**	**P-value****	**1**	**10**	**P-value****
**0.3**	99.4 (0.04)	99.1 (0.04)	0.757	82.1 (0.9)	80.2 (1.1)	0.699
**0.5**	99.0 (0.07)	99.4 (0.04)		80.3 (1.6)	83.4 (1.0)	
**0.7**	99.5 (0.26)	99.1 (0.13)		92.5 (0.5)	94.3 (0.2)	
**1**	99.7 (0.05)	99.5 (0.05)		93.2 (0.4)	95.2 (0.3)	
**2**	99.7 (0.06)	99.7 (0.04)		97.5 (0.3)	98.5 (0.1)	
**5**	99.8 (0.15)	99.9 (0.11)		99.2 (0.2)	99.6 (0.4)	

*The sample size for 1 minute and 10 minutes were 2.8 and 28 liters of air respectively.
** Based on One Way Repeated Measures Analysis of Variance analysis.

Supplementary Material 3: The raw data of filtration efficiency (FE) of FFRs.

Type: 3M-8210				Date: 27/08/2020						
		Control			KD					
Particle Size (uM)	Mask#	Particle#	Average	Standrad error	Particle#	Average	Standrad error	FE (Norm. to PC %)	Average%	Standrad error
0.3	1	37791.0	35266.2	823.5	6560.0	7025.8	350.8	81.4	80.1	1.0
	2	37335.0			7750.0			78.0		
	3	35826.0			8478.0			76.0		
	4	33992.0			6350.0			82.0		
	5	33666.0			5561.0			84.2		
	6	32987.0			5390.0			84.7		
	7				9601.0			72.8		
	8				6850.0			80.6		
	9				7081.0			79.9		
	10				6385.0			81.9		
	11				6443.0			81.7		
	12				7861.0			77.7		
0.5	1	3729.0	3079.8	278.1	265.0	481.1	105.3	91.4	84.4	3.4
	2	3896.0			1218.0			60.5		
	3	3303.0			378.0			87.7		
	4	2855.0			910.0			70.5		
	5	2522.0			220.0			92.9		
	6	2174.0			195.0			93.7		
	7				1083.0			64.8		
	8				265.0			91.4		
	9				269.0			91.3		
	10				325.0			89.4		
	11				335.0			89.1		
	12				310.0			89.9		
0.7	1	**153.0**	130.7	11.7	15.0	13.1	1.7	88.5	90.0	1.3
	2	175.0			25.0			80.9		
	3	121.0			15.0			88.5		
	4	129.0			13.0			90.1		
	5	106.0			7.0			94.6		
	6	100.0			13.0			90.1		
	7				22.0			83.2		
	8				5.0			96.2		
	9				12.0			90.8		
	10				6.0			95.4		
	11				10.0			92.3		
	12				14.0			89.3		
1.0	1	**280.0**	276.5	13.6	23.0	19.6	2.2	91.7	92.9	0.8
	2	334.0			33.0			88.1		
	3	289.0			20.0			92.8		
	4	264.0			22.0			92.0		
	5	250.0			12.0			95.7		
	6	242.0			13.0			95.3		
	7				22.0			92.0		
	8				7.0			97.5		
	9				20.0			92.8		
	10				18.0			93.5		
	11				14.0			94.9		
	12				31.0			88.8		
2.0	1	**138.0**	145.0	6.7	2.0	4.4	0.7	98.6	97.0	0.5
	2	154.0			5.0			96.6		
	3	131.0			7.0			95.2		
	4	145.0			5.0			96.6		
	5	129.0			7.0			95.2		
	6	173.0			6.0			95.9		
	7				4.0			97.2		
	8				1.0			99.3		
	9				1.0			99.3		
	10				7.0			95.2		
	11				2.0			98.6		
	12				6.0			95.9		
5.0	1	**65.0**	60.5	1.8	1.0	0.4	0.2	98.3	99.3	0.3
	2	61.0			0.0			100.0		
	3	59.0			0.0			100.0		
	4	56.0			0.0			100.0		
	5	56.0			2.0			96.7		
	6	66.0			0.0			100.0		
	7				1.0			98.3		
	8				0.0			100.0		
	9				0.0			100.0		
	10				1.0			98.3		
	11				0.0			100.0		
	12				0.0			100.0		

Gerson 1730 N95 Mask				Date: 12/10/2020						
		Control			KD					
Particle Size (uM)	Mask#	Particle#	Average	Standrad error	Particle#	Average	Standrad error	FE (Norm. to PC %)	Average%	Standrad error
	1	64874.0			482.0			99.2		
	2	63908.0			285.0			99.6		
	3	65191.0			181.0			99.7		
	4	64113.0			332.0			99.5		
	5	64051.0			563.0			99.1		
0.3	6	62941.0	64179.7	323.2		368.6	68.6		99.4	0.1
	7									
	8									
	9									
	10									
	11									
	12									
	1	4607.0			29.0			99.4		
	2	4516.0			23.0			99.5		
	3	4588.0			41.0			99.1		
	4	4383.0			35.0			99.2		
	5	4500.0			61.0			98.6		
0.5	6	4392.0	4497.7	38.6		37.8	6.5		99.2	0.1
	7									
	8									
	9									
	10									
	11									
	12									
	1	112.0			0.0			100.0		
	2	106.0			1.0			99.2		
	3	145.0			0.0			100.0		
	4	106.0			0.0			100.0		
	5	139.0			1.0			99.2		
0.7	6	109.0	119.5	7.2		0.4	0.2		99.7	0.2
	7									
	8									
	9									
	10									
	11									
	12									
	1	276.0			0.0			100.0		
	2	268.0			1.0			99.6		
	3	260.0			1.0			99.6		
	4	260.0			1.0			99.6		
	5	242.0			6.0			97.7		
1.0	6	255.0	260.2	4.7		1.8	1.1		99.3	0.4
	7									
	8									
	9									
	10									
	11									
	12									
	1	137.0			0.0			100.0		
	2	165.0			1.0			99.4		
	3	168.0			0.0			100.0		
	4	184.0			0.0			100.0		
	5	147.0			3.0			98.1		
2.0	6	164.0	160.8	6.8		0.8	0.6		99.5	0.4
	7									
	8									
	9									
	10									
	11									
	12									
	1	37.0			0.0			100.0		
	2	39.0			0.0			100.0		
	3	40.0			0.0			100.0		
	4	48.0			0.0			100.0		
	5	45.0			1.0			97.6		
5.0	6	43.0	42.0	1.7		0.2	0.2		99.5	0.5
	7									
	8									
	9									
	10									
	11									
	12									

Medline N95 Cone Style Masks				Date: 12/10/2020						
		Control			KD					
Particle Size (uM)	Mask#	Particle#	Average	Standrad error	Particle#	Average	Standrad error	FE (Norm. to PC %)	Average%	Standrad error
0.3	1	64874.0	64179.7	323.2	42.0	49.2	4.1	99.9	99.9	0.0
	2	63908.0			53.0			99.9		
	3	65191.0			37.0			99.9		
	4	64113.0			58.0			99.9		
	5	64051.0			56.0			99.9		
	6	62941.0								
	7									
	8									
	9									
	10									
	11									
	12									
0.5	1	4607.0	4497.7	38.6	4.0	5.6	1.7	99.9	99.9	0.0
	2	4516.0			11.0			99.8		
	3	4588.0			7.0			99.8		
	4	4383.0			5.0			99.9		
	5	4500.0			1.0			100.0		
	6	4392.0								
	7									
	8									
	9									
	10									
	11									
	12									
0.7	1	112.0	119.5	7.2	1.0	0.6	0.2	99.2	99.5	0.2
	2	106.0			0.0			100.0		
	3	145.0			1.0			99.2		
	4	106.0			1.0			99.2		
	5	139.0			0.0			100.0		
	6	109.0								
	7									
	8									
	9									
	10									
	11									
	12									
1.0	1	276.0	260.2	4.7	1.0	1.6	0.4	99.6	99.4	0.2
	2	268.0			1.0			99.6		
	3	260.0			2.0			99.2		
	4	260.0			3.0			98.8		
	5	242.0			1.0			99.6		
	6	255.0								
	7									
	8									
	9									
	10									
	11									
	12									
2.0	1	137.0	160.8	6.8	3.0	3.0	0.8	98.1	98.1	0.5
	2	165.0			5.0			96.9		
	3	168.0			0.0			100.0		
	4	184.0			4.0			97.5		
	5	147.0			3.0			98.1		
	6	164.0								
	7									
	8									
	9									
	10									
	11									
	12									
5.0	1	37.0	42.0	1.7	0.0	0.8	0.6	100.0	98.1	1.4
	2	39.0			0.0			100.0		
	3	40.0			0.0			100.0		
	4	48.0			3.0			92.9		
	5	45.0			1.0			97.6		
	6	43.0								
	7									
	8									
	9									
	10									
	11									
	12									

Benehal N95 mask	Lot: Number			Date: 11/10/2020						
		Control			KD					
Particle Size (uM)	Mask#	Particle#	Average	Standrad error	Particle#	Average	Standrad error	FE (Norm. to PC %)	Average%	Standrad error
0.3	1	71471.0	69255.8	1163.7	10153.0	9571.8	2485.4	85.3	86.2	3.6
	2	70682.0			17767.0			74.3		
	3	68592.0			8285.0			88.0		
	4	66278.0			2198.0			96.8		
	5				9456.0			86.3		
	6									
	7									
	8									
	9									
	10									
	11									
	12									
0.5	1	9392.0	8321.8	541.3	855.0	701.8	176.4	89.7	91.6	2.1
	2	9029.0			1238.0			85.1		
	3	7814.0			596.0			92.8		
	4	7052.0			155.0			98.1		
	5				665.0			92.0		
	6									
	7									
	8									
	9									
	10									
	11									
	12									
0.7	1	359.0	303.0	30.2	23.0	25.6	3.6	92.4	91.6	1.2
	2	340.0			34.0			88.8		
	3	289.0			16.0			94.7		
	4	224.0			34.0			88.8		
	5				21.0			93.1		
	6									
	7									
	8									
	9									
	10									
	11									
	12									
1.0	1	492.0	459.3	18.0	42.0	37.8	3.9	90.9	91.8	0.9
	2	476.0			25.0			94.6		
	3	460.0			34.0			92.6		
	4	409.0			40.0			91.3		
	5				48.0			89.5		
	6									
	7									
	8									
	9									
	10									
	11									
	12									
2.0	1	164.0	201.8	15.4	15.0	15.4	3.5	92.6	92.4	1.8
	2	239.0			17.0			91.6		
	3	198.0			13.0			93.6		
	4	206.0			5.0			97.5		
	5				27.0			86.6		
	6									
	7									
	8									
	9									
	10									
	11									
	12									
5.0	1	27.0	35.0	2.8	1.0	2.0	0.4	97.1	94.3	1.3
	2	35.0			2.0			94.3		
	3	39.0			3.0			91.4		
	4	39.0			3.0			91.4		
	5				1.0			97.1		
	6									
	7									
	8									
	9									
	10									
	11									
	12									

N99/N95 SpectraShield Plus Mask				Date: 11/10/2020						
		Control			KD					
Particle Size (uM)	Mask#	Particle#	Average	Standrad error	Particle#	Average	Standrad error	FE (Norm. to PC %)	Average%	Standrad error
0.3	1	71471.0	69255.8	1163.7	205.0	318.8	38.0	99.7	99.5	0.1
	2	70682.0			403.0			99.4		
	3	68592.0			351.0			99.5		
	4	66278.0			380.0			99.5		
	5				255.0			99.6		
	6									
	7									
	8									
	9									
	10									
	11									
	12									
0.5	1	9392.0	8321.8	541.3	21.0	28.4	2.2	99.7	99.7	0.0
	2	9029.0			34.0			99.6		
	3	7814.0			32.0			99.6		
	4	7052.0			28.0			99.7		
	5				27.0			99.7		
	6									
	7									
	8									
	9									
	10									
	11									
	12									
0.7	1	359.0	303.0	30.2	0.0	0.4	0.4	100.0	99.9	0.1
	2	340.0			2.0			99.3		
	3	289.0			0.0			100.0		
	4	224.0			0.0			100.0		
	5				0.0			100.0		
	6									
	7									
	8									
	9									
	10									
	11									
	12									
1.0	1	492.0	459.3	18.0	2.0	1.6	0.4	99.6	99.7	0.1
	2	476.0			2.0			99.6		
	3	460.0			2.0			99.6		
	4	409.0			2.0			99.6		
	5				0.0			100.0		
	6									
	7									
	8									
	9									
	10									
	11									
	12									
2.0	1	164.0	201.8	15.4	2.0	1.2	0.4	99.0	99.4	0.2
	2	239.0			0.0			100.0		
	3	198.0			1.0			99.5		
	4	206.0			2.0			99.0		
	5				1.0			99.5		
	6									
	7									
	8									
	9									
	10									
	11									
	12									
5.0	1	27.0	35.0	2.8	1.0	0.2	0.2	97.1	99.4	0.6
	2	35.0			0.0			100.0		
	3	39.0			0.0			100.0		
	4	39.0			0.0			100.0		
	5				0.0			100.0		
	6									
	7									
	8									
	9									
	10									
	11									
	12									

KN95 (Duck shape)				Date: 12/10/2020						
		Control			KD					
Particle Size (uM)	Mask#	Particle#	Average	Standrad error	Particle#	Average	Standrad error	FE (Norm. to PC %)	Average%	Standrad error
0.3	1	51219.0	49660.0	526.2	1086.0	675.6	197.9	97.8	98.6	0.4
	2	50338.0			1045.0			97.9		
	3	48366.0			395.0			99.2		
	4	49703.0			797.0			98.4		
	5	48674.0			55.0			99.9		
	6									
	7									
	8									
	9									
	10									
	11									
	12									
0.5	1	5990.0	5647.4	127.8	103.0	49.2	16.7	98.2	99.1	0.3
	2	5924.0			67.0			98.8		
	3	5477.0			19.0			99.7		
	4	5461.0			46.0			99.2		
	5	5385.0			11.0			99.8		
	6									
	7									
	8									
	9									
	10									
	11									
	12									
0.7	1	271.0	237.4	14.5	4.0	1.8	0.8	98.3	99.2	0.3
	2	219.0			3.0			98.7		
	3	225.0			0.0			100.0		
	4	272.0			0.0			100.0		
	5	200.0			2.0			99.2		
	6									
	7									
	8									
	9									
	10									
	11									
	12									
1.0	1	360.0	363.4	4.0	2.0	1.6	0.7	99.4	99.6	0.2
	2	375.0			4.0			98.9		
	3	351.0			0.0			100.0		
	4	363.0			2.0			99.4		
	5	368.0			0.0			100.0		
	6									
	7									
	8									
	9									
	10									
	11									
	12									
2.0	1	138.0	122.8	5.2	0.0	0.2	0.2	100.0	99.8	0.2
	2	123.0			0.0			100.0		
	3	127.0			0.0			100.0		
	4	106.0			1.0			99.2		
	5	120.0			0.0			100.0		
	6									
	7									
	8									
	9									
	10									
	11									
	12									
5.0	1	17.0	19.2	1.5	0.0	0.0	0.0	100.0	100.0	0.0
	2	19.0			0.0			100.0		
	3	23.0			0.0			100.0		
	4	15.0			0.0			100.0		
	5	22.0			0.0			100.0		
	6									
	7									
	8									
	9									
	10									
	11									
	12									

Supplementary Material 4: The raw data of filtration efficiency (FE) stability of N95-8210 and KN95 masks following VHP-STERIS Sterilization Systems.

Type: 3M-8210																		
	Cycle 1									Cycle 2								
	Control			KD						Control			KD					
Particle Size (uM)	Particle #	Average	Standrad error	Particle#	Average	Standrad error	FE (Norm. to cycle 0)	Average%	Standrad error	Particle #	Average	Standrad error	Particle#	Average	Standrad error	FE (Norm. to cycle 0)	Average%	Standrad error
	35193.0			6553.0			96.6			20065.0			4405.0			90.2		
	34476.0			6501.0			96.9			16317.0			4149.0			92.2		
	27402.0			6775.0			95.7			15983.0			3486.0			97.4		
	26748.0			5453.0			101.4			15327.0			3101.0			100.4		
	23924.0			6053.0			98.8			15753.0			6120.0			76.7		
0.3	26215.0	28993.0	1910.4	8277.0	6475.5	299.3	89.2	97.0	1.3	15267.0	15862.9	492.3	3267.0	4023.8	341.7	99.1	93.2	2.7
				6275.0			97.8			15035.0			2585.0			104.5		
				5933.0			99.3			14731.0			3266.0			99.1		
				6579.0			96.5			14890.0			3451.0			97.7		
				5553.0			100.9			15261.0			3674.0			95.9		
				8571.0			87.9						4316.0			90.9		
				5183.0			102.5						6466.0			74.0		
	2250.0			1271.0			41.4			2454.0			451.0			90.6		
	2024.0			478.0			89.5			1836.0			446.0			90.9		
	1693.0			476.0			89.6			2015.0			357.0			96.4		
	1646.0			306.0			99.9			1932.0			327.0			98.3		
	1514.0			368.0			96.2			1909.0			872.0			64.6		
0.5	2590.0	1952.8	168.7	468.0	459.8	77.6	90.1	90.6	4.7	1905.0	1918.1	65.7	900.0	507.5	79.5	62.9	87.1	4.9
				344.0			97.6			1845.0			135.0			110.1		
				307.0			99.9			1716.0			357.0			96.4		
				468.0			90.1			1749.0			320.0			98.7		
				213.0			105.6			1820.0			388.0			94.5		
				378.0			95.5						506.0			87.2		
				441.0			91.7						1031.0			54.8		
	133.0			25.0			87.7			201.0			27.0			93.3		
	131.0			11.0			100.8			172.0			32.0			90.0		
	91.0			15.0			97.1			175.0			20.0			97.9		
	98.0			9.0			102.7			164.0			46.0			80.8		
	108.0			13.0			99.0			186.0			20.0			97.9		
0.7	152.0	118.8	9.6	20.0	16.3	2.0	92.4	95.9	1.9	161.0	168.4	5.1	22.0	28.1	2.8	96.6	92.6	1.9
				16.0			96.2			154.0			19.0			98.6		
				7.0			104.6			144.0			20.0			97.9		
				23.0			89.6			164.0			20.0			97.9		
				14.0			98.0			163.0			30.0			91.3		
				30.0			83.1						36.0			87.4		
				12.0			99.9						45.0			81.4		
	357.0			76.0			86.1			605.0			78.0			91.9		
	343.0			35.0			97.7			511.0			75.0			92.5		
	264.0			37.0			97.2			618.0			53.0			96.9		
	328.0			26.0			100.3			529.0			40.0			99.5		
	440.0			31.0			98.9			568.0			34.0			100.8		
1.0	551.0	380.5	41.2	42.0	42.8	5.3	95.8	95.5	1.5	473.0	531.9	15.9	48.0	62.4	5.4	97.9	95.0	1.1
				48.0			94.1			528.0			85.0			90.4		
				32.0			98.6			481.0			47.0			98.1		
				80.0			85.0			486.0			52.0			97.1		
				27.0			100.0			520.0			82.0			91.0		
				50.0			93.5						87.0			90.0		
				29.0			99.4						68.0			93.9		
	357.0			22.0			97.1			333.0			18.0			98.1		
	343.0			3.0			102.3			407.0			31.0			94.5		
	264.0			19.0			97.9			473.0			16.0			98.7		
	328.0			29.0			95.2			399.0			9.0			100.6		
	440.0			22.0			97.1			442.0			10.0			100.3		
2.0	551.0	380.5	41.2	16.0	16.2	2.2	98.8	98.7	0.6	399.0	371.9	18.9	20.0	18.8	2.1	97.5	97.9	0.6
				11.0			100.1			325.0			28.0			95.3		
				10.0			100.4			326.0			14.0			99.2		
				8.0			100.9			312.0			11.0			100.0		
				11.0			100.1			303.0			20.0			97.5		
				22.0			97.1						23.0			96.7		
				21.0			97.4						25.0			96.2		
	42.0			2.0			98.6			38.0			2.0			97.8		
	48.0			0.0			100.7			73.0			0.0			100.7		
	30.0			2.0			98.6			106.0			2.0			97.8		
	65.0			1.0			99.6			88.0			2.0			97.8		
	183.0			4.0			96.4			69.0			0.0			100.7		
5.0	199.0	94.5	30.9	1.0	1.7	0.3	99.6	98.9	0.4	73.0	68.7	5.9	1.0	1.0	0.2	99.2	99.2	0.4
				1.0			99.6			59.0			1.0			99.2		
				1.0			99.6			59.0			0.0			100.7		
				3.0			97.5			55.0			1.0			99.2		
				1.0			99.6			67.0			1.0			99.2		
				3.0			97.5						2.0			97.8		
				1.0			99.6						0.0			100.7		

Type: 3M-8210

Cycle 3

Particle Size (uM)	Control Particle#	Control Average	Control Standrad error	KD Particle#	KD Average	KD Standrad error	FE (Norm. to cycle 0)	Average%	Standrad error
0.3	14207.0	13726.3	199.0	5475.0	4104.2	297.2	75.0	87.5	2.7
	13596.0			2700.0			100.3		
	13012.0			5188.0			77.7		
	13852.0			5476.0			75.0		
	13483.0			4065.0			87.9		
	13084.0			4878.0			80.5		
	13685.0			4370.0			85.1		
	13367.0			2554.0			101.6		
	13773.0			3066.0			97.0		
	15204.0			3679.0			91.4		
				4309.0			85.7		
				3490.0			93.1		
0.5	1591.0	1491.7	45.4	450.0	414.5	35.2	82.7	85.6	2.8
	1543.0			459.0			82.0		
	1419.0			580.0			72.4		
	1501.0			250.0			98.6		
	1426.0			684.0			64.2		
	1372.0			369.0			89.2		
	1371.0			396.0			87.0		
	1392.0			263.0			97.6		
	1459.0			400.0			86.7		
	1843.0			351.0			90.6		
				356.0			90.2		
				416.0			85.4		
0.7	136.0	102.5	4.8	29.0	20.3	1.8	79.7	89.1	1.9
	94.0			12.0			98.1		
	98.0			22.0			87.3		
	111.0			13.0			97.0		
	93.0			15.0			94.9		
	96.0			29.0			79.7		
	83.0			20.0			89.4		
	92.0			18.0			91.6		
	107.0			19.0			90.5		
	115.0			15.0			94.9		
				28.0			80.8		
				24.0			85.1		
1.0	439.0	370.1	11.4	37.0	50.3	3.1	96.9	93.0	0.9
	364.0			53.0			92.2		
	361.0			58.0			90.8		
	387.0			34.0			97.8		
	418.0			45.0			94.6		
	316.0			43.0			95.1		
	337.0			55.0			91.6		
	359.0			42.0			95.4		
	359.0			49.0			93.4		
	361.0			55.0			91.6		
				64.0			89.0		
				69.0			87.6		
2.0	494.0	376.1	17.1	22.0	18.8	1.6	97.1	98.0	0.4
	422.0			17.0			98.4		
	354.0			17.0			98.4		
	352.0			25.0			96.2		
	383.0			25.0			96.2		
	327.0			23.0			96.8		
	305.0			21.0			97.3		
	344.0			14.0			99.3		
	399.0			5.0			101.7		
	381.0			20.0			97.6		
				15.0			99.0		
				21.0			97.3		
5.0	115.0	108.9	4.8	1.0	0.4	0.1	99.8	100.3	0.1
	131.0			1.0			99.8		
	102.0			1.0			99.8		
	129.0			0.0			100.7		
	121.0			0.0			100.7		
	95.0			0.0			100.7		
	95.0			0.0			100.7		
	87.0			0.0			100.7		
	112.0			0.0			100.7		
	102.0			1.0			99.8		
				1.0			99.8		
				0.0			100.7		

Cycle 4

Particle Size (uM)	Control Particle#	Control Particle#	Control Standrad error	KD Particle#	KD Average	KD Standrad error	FE (Norm. to cycle 0)	Average%	Standrad error
0.3	24952.0	25638.7	328.7	9633.0	9545.5	352.1	77.9	78.4	1.7
	25467.0			8563.0			83.1		
	25707.0			11210.0			70.3		
	25545.0			9866.0			76.8		
	24468.0			7852.0			86.6		
	26145.0			7890.0			86.4		
	27187.0			9565.0			78.3		
				8963.0			81.2		
				12023.0			66.3		
				9963.0			76.3		
				9863.0			76.8		
				9155.0			80.3		
0.5	1475.0	1651.4	158.3	393.0	466.8	29.5	90.3	85.0	2.1
	1505.0			407.0			89.3		
	1507.0			386.0			90.8		
	1397.0			334.0			94.5		
	1490.0			600.0			75.4		
	1676.0			330.0			94.8		
	2740.0			387.0			90.7		
	1421.0			541.0			79.7		
				554.0			78.7		
				550.0			79.0		
				588.0			76.3		
				531.0			80.4		
0.7	100.0	101.1	8.0	29.0	19.2	2.3	79.2	90.1	2.6
	98.0			20.0			89.1		
	110.0			17.0			92.4		
	96.0			8.0			102.3		
	88.0			9.0			101.2		
	86.0			10.0			100.1		
	152.0			21.0			88.0		
	79.0			15.0			94.6		
				25.0			83.6		
				26.0			82.5		
				33.0			74.9		
				17.0			92.4		
1.0	351.0	330.6	11.5	29.0	46.7	6.0	98.2	92.4	1.9
	353.0			80.0			81.6		
	366.0			40.0			94.6		
	319.0			32.0			97.2		
	355.0			85.0			80.0		
	325.0			25.0			99.5		
	308.0			30.0			97.9		
	268.0			27.0			98.9		
				47.0			92.3		
				44.0			93.3		
				63.0			87.1		
				58.0			88.8		
2.0	468.0	373.5	30.3	16.0	16.3	1.8	98.7	98.6	0.5
	461.0			32.0			94.3		
	437.0			14.0			99.2		
	341.0			13.0			99.5		
	397.0			9.0			100.6		
	376.0			24.0			96.5		
	235.0			13.0			99.5		
	273.0			11.0			100.1		
				13.0			99.5		
				14.0			99.2		
				19.0			97.8		
				18.0			98.1		
5.0	124.0	107.9	10.1	0.0	0.6	0.1	100.7	100.2	0.1
	130.0			1.0			99.8		
	117.0			0.0			100.7		
	111.0			0.0			100.7		
	125.0			1.0			99.8		
	124.0			1.0			99.8		
	46.0			0.0			100.7		
	86.0			0.0			100.7		
				1.0			99.8		
				1.0			99.8		
				1.0			99.8		
				1.0			99.8		

Type: 3M-8210																		
	**Cycle 5**									**Cycle 6**								
	Control			KD						Control			KD					
Particle Size (uM)	Particle #	Average	Standard error	Particle#	Average	Standard error	FE (Norm. to cycle 0)	Average %	Standard error	Particle #	Average	Standard error	Particle#	Average	Standard error	FE (Norm. to cycle 0)	Average %	Standrad error
0.3	16031.0			6536.0			72.9			10484.0			4779.0			68.9		
	15957.0			6236.0			75.3			10891.0			4372.0			73.7		
	15718.0			6486.0			73.3			11238.0			4001.0			78.0		
	15446.0			5995.0			77.2			10693.0			4437.0			72.9		
	15570.0			5666.0			79.8			10566.0			4292.0			74.6		
	15555.0	15712.8	96.2	5442.0	6134.8	165.1	81.6	76.1	1.3	10113.0	10664.2	155.7	4155.0	4282.9	71.5	76.2	74.7	0.8
				5025.0			84.9						4686.0			70.0		
				5952.0			77.6						4264.0			74.9		
				6175.0			75.8						4070.0			77.2		
				7025.0			69.0						4075.0			77.1		
				6855.0			70.4						4179.0			75.9		
				6225.0			75.4						4085.0			77.0		
0.5	1181.0			395.0			79.8			1374.0			365.0			86.7		
	1170.0			370.0			82.3			1493.0			348.0			88.2		
	1167.0			355.0			83.7			1401.0			306.0			91.8		
	1227.0			363.0			83.0			1357.0			378.0			85.5		
	1327.0			322.0			87.0			1345.0			363.0			86.8		
	1192.0	1210.7	24.9	310.0	349.2	7.1	88.1	84.3	0.7	1187.0	1359.5	40.7	367.0	416.8	27.9	86.5	82.2	2.4
				348.0			84.4						418.0			82.1		
				329.0			86.3						403.0			83.4		
				338.0			85.4						669.0			60.2		
				380.0			81.3						478.0			76.8		
				341.0			85.1						507.0			74.3		
				339.0			85.3						400.0			83.6		
0.7	83.0			13.0			95.8			104.0			21.0			88.4		
	84.0			34.0			71.0			99.0			22.0			87.3		
	86.0			24.0			82.8			112.0			20.0			89.5		
	100.0			29.0			76.9			110.0			28.0			80.9		
	116.0			12.0			97.0			91.0			21.0			88.4		
	96.0	94.2	5.2	25.0	19.4	2.2	81.6	88.2	2.6	101.0	102.8	3.1	26.0	21.9	1.1	83.0	87.4	1.2
				15.0			93.4						22.0			87.3		
				15.0			93.4						24.0			85.2		
				16.0			92.2						23.0			86.3		
				14.0			94.6						25.0			84.1		
				10.0			99.3						15.0			94.9		
				26.0			80.4						16.0			93.8		
1.0	313.0			40.0			94.7			338.0			40.0			94.4		
	313.0			62.0			87.6			367.0			45.0			92.8		
	321.0			41.0			94.4			327.0			46.0			92.4		
	297.0			67.0			86.0			316.0			48.0			91.8		
	364.0			45.0			93.1			295.0			30.0			97.7		
	387.0	332.5	14.3	65.0	46.1	4.9	86.6	92.7	1.6	311.0	325.7	10.2	49.0	47.6	2.7	91.4	91.9	0.9
				33.0			97.0						35.0			96.1		
				26.0			99.2						48.0			91.8		
				23.0			100.2						53.0			90.1		
				47.0			92.4						59.0			88.1		
				31.0			97.6						58.0			88.5		
				73.0			84.0						60.0			87.8		
2.0	256.0			17.0			96.5			200.0			10.0			98.0		
	224.0			16.0			96.9			244.0			15.0			95.4		
	260.0			11.0			98.8			212.0			10.0			98.0		
	235.0			19.0			95.8			201.0			8.0			99.0		
	296.0			3.0			101.9			196.0			7.0			99.5		
	330.0	266.8	16.2	13.0	11.6	1.7	98.1	98.6	0.7	154.0	201.2	11.8	9.0	10.0	1.1	98.5	98.0	0.6
				8.0			100.0						10.0			98.0		
				4.0			101.5						6.0			100.0		
				8.0			100.0						14.0			95.9		
				20.0			95.4						5.0			100.5		
				5.0			101.2						18.0			93.9		
				15.0			97.3						8.0			99.0		
5.0	47.0			1.0			98.8			25.0			0.0			100.7		
	48.0			1.0			98.8			20.0			0.0			100.7		
	36.0			0.0			100.7			24.0			0.0			100.7		
	63.0			0.0			100.7			23.0			0.0			100.7		
	61.0			0.0			100.7			29.0			0.0			100.7		
	62.0	52.8	4.5	0.0	0.3	0.1	100.7	100.2	0.2	28.0	24.8	1.4	0.0	0.2	0.1	100.7	100.0	0.5
				0.0			100.7						0.0			100.7		
				0.0			100.7						0.0			100.7		
				0.0			100.7						1.0			96.6		
				0.0			100.7						0.0			100.7		
				0.0			100.7						0.0			100.7		
				1.0			98.8						1.0			96.6		

Type: 3M-8210

Cycle 7

Particle Size (uM)	Control Particle#	Control Average	Control Standra d error	KD Particle#	KD Average	KD Standra d error	FE (Norm. to cycle 0)	Average %	Standrad error
0.3	21301.0	22187.8	198.0	10057.0	8393.8	242.0	68.3	77.6	1.4
	22082.0			8208.0			78.7		
	22616.0			7728.0			81.4		
	22503.0			8455.0			77.3		
	22493.0			7095.0			84.9		
	22132.0			9626.0			70.7		
				8166.0			78.9		
				8003.0			79.8		
				8688.0			76.0		
				7456.0			82.9		
				8604.0			76.4		
				8640.0			76.2		
0.5	1677.0	1646.2	18.5	573.0	480.2	17.4	77.2	83.9	1.3
	1567.0			467.0			84.9		
	1663.0			419.0			88.3		
	1694.0			477.0			84.2		
	1652.0			415.0			88.6		
	1624.0			587.0			76.2		
				526.0			80.6		
				454.0			85.8		
				506.0			82.1		
				390.0			90.4		
				476.0			84.2		
				472.0			84.5		
0.7	104.0	120.3	6.4	23.0	25.3	1.3	89.9	87.8	1.2
	101.0			24.0			89.0		
	126.0			29.0			84.3		
	143.0			21.0			91.7		
	124.0			19.0			93.6		
	124.0			22.0			90.8		
				27.0			86.2		
				25.0			88.0		
				31.0			82.5		
				33.0			80.6		
				29.0			84.3		
				20.0			92.6		
1.0	380.0	386.2	4.5	50.0	55.4	1.1	93.7	92.2	0.3
	385.0			56.0			92.0		
	397.0			54.0			92.6		
	390.0			53.0			92.9		
	397.0			59.0			91.2		
	368.0			58.0			91.5		
				59.0			91.2		
				50.0			93.7		
				59.0			91.2		
				52.0			93.1		
				54.0			92.6		
				61.0			90.6		
2.0	243.0	270.0	9.4	19.0	11.4	1.2	95.8	98.7	0.5
	278.0			14.0			97.7		
	309.0			15.0			97.4		
	274.0			17.0			96.6		
	255.0			12.0			98.5		
	261.0			10.0			99.3		
				9.0			99.7		
				9.0			99.7		
				11.0			98.9		
				10.0			99.3		
				5.0			101.2		
				6.0			100.8		
5.0	25.0	30.8	2.7	0.0	0.3	0.1	100.7	99.9	0.4
	33.0			0.0			100.7		
	35.0			0.0			100.7		
	41.0			1.0			97.4		
	25.0			0.0			100.7		
	26.0			1.0			97.4		
				0.0			100.7		
				0.0			100.7		
				0.0			100.7		
				0.0			100.7		
				1.0			97.4		
				0.0			100.7		

Cycle 8

Particle Size (uM)	Control Particle #	Control Average	Control Standra d error	KD Particle#	KD Average	KD Standra d error	FE (Norm. to cycle 0)	Average %	Standra d error
0.3	45828.0	45457.2	285.5	18025.0	17864.1	259.2	75.3	75.8	0.7
	45720.0			18563.0			73.9		
	45812.0			19001.0			72.7		
	45329.0			18359.0			74.4		
	45956.0			19057.0			72.5		
	44098.0			17256.0			77.5		
				17896.0			75.7		
				16025.0			80.8		
				17856.0			75.8		
				17896.0			75.7		
				17850.0			75.8		
				16585.0			79.3		
0.5	3603.0	3777.2	78.6	1026.0	1122.4	19.7	86.3	83.3	0.6
	3513.0			1147.0			82.5		
	3899.0			1148.0			82.5		
	3981.0			1168.0			81.8		
	3940.0			1123.0			83.3		
	3727.0			1199.0			80.9		
				1025.0			86.3		
				1190.0			81.2		
				1147.0			82.5		
				1189.0			81.2		
				1009.0			86.8		
				1098.0			84.0		
0.7	183.0	187.5	13.9	36.0	38.9	1.3	89.8	88.0	0.8
	160.0			42.0			86.2		
	236.0			45.0			84.4		
	223.0			36.0			89.8		
	166.0			39.0			88.0		
	157.0			42.0			86.2		
				37.0			89.2		
				35.0			90.4		
				36.0			89.8		
				35.0			90.4		
				35.0			90.4		
				49.0			82.1		
1.0	482.0	514.8	15.1	67.0	73.8	2.2	93.6	92.2	0.5
	488.0			70.0			93.0		
	575.0			75.0			92.0		
	534.0			79.0			91.1		
	526.0			70.0			93.0		
	484.0			65.0			94.1		
				81.0			90.7		
				62.0			94.7		
				69.0			93.2		
				80.0			90.9		
				81.0			90.7		
				86.0			89.7		
2.0	465.0	458.7	22.9	20.0	21.4	1.4	98.6	98.3	0.3
	524.0			15.0			99.7		
	494.0			21.0			98.4		
	473.0			23.0			97.9		
	434.0			29.0			96.6		
	362.0			27.0			97.0		
				26.0			97.2		
				26.0			97.2		
				19.0			98.8		
				15.0			99.7		
				18.0			99.0		
				18.0			99.0		
5.0	158.0	139.7	12.6	1.0	0.8	0.2	100.0	100.2	0.2
	176.0			1.0			100.0		
	153.0			0.0			100.7		
	143.0			0.0			100.7		
	118.0			1.0			100.0		
	90.0			1.0			100.0		
				0.0			100.7		
				0.0			100.7		
				0.0			100.7		
				2.0			99.3		
				2.0			99.3		
				1.0			100.0		

Type: 3M-8210

Cycle 9

Particle Size (uM)	Control: Particle #	Control: Average	Control: Standrad error	KD: Particle#	KD: Average	KD: Standrad error	KD: FE (Norm. to cycle 0)	KD: Average %	KD: Standrad error
0.3	57832.0	57145.7	615.4	23025.0	22918.7	570.8	74.5	74.8	1.2
	59334.0			23345.0			73.8		
	58159.0			21589.0			77.7		
	56001.0			25987.0			68.1		
	55797.0			23657.0			73.2		
	55751.0			20369.0			80.3		
				21479.0			77.9		
				19203.0			82.9		
				23658.0			73.2		
				23487.0			73.5		
				25636.0			68.8		
				23589.0			73.3		
0.5	4491.0	4439.3	84.4	1309.0	1344.6	22.6	83.5	82.6	0.6
	4604.0			1412.0			80.8		
	4689.0			1329.0			83.0		
	4458.0			1225.0			85.8		
	4240.0			1203.0			86.4		
	4154.0			1435.0			80.2		
				1356.0			82.3		
				1326.0			83.1		
				1335.0			82.9		
				1420.0			80.6		
				1456.0			79.6		
				1329.0			83.0		
0.7	164.0	162.0	4.7	39.0	33.9	1.2	84.4	87.8	0.8
	145.0			35.0			87.1		
	168.0			30.0			90.5		
	166.0			29.0			91.2		
	177.0			35.0			87.1		
	152.0			39.0			84.4		
				36.0			86.4		
				30.0			90.5		
				37.0			85.7		
				26.0			93.3		
				36.0			86.4		
				35.0			87.1		
1.0	581.0	672.5	23.3	99.0	97.9	1.9	91.8	92.0	0.3
	639.0			91.0			93.1		
	668.0			94.0			92.6		
	697.0			86.0			93.9		
	707.0			96.0			92.3		
	743.0			101.0			91.5		
				106.0			90.7		
				91.0			93.1		
				109.0			90.2		
				102.0			91.3		
				98.0			92.0		
				102.0			91.3		
2.0	613.0	698.8	18.3	37.0	32.9	1.6	97.6	98.2	0.2
	689.0			30.0			98.7		
	716.0			22.0			99.8		
	733.0			42.0			96.9		
	729.0			35.0			97.9		
	713.0			37.0			97.6		
				36.0			97.8		
				37.0			97.6		
				32.0			98.4		
				33.0			98.2		
				28.0			99.0		
				26.0			99.3		
5.0	120.0	157.0	10.5	1.0	1.1	0.5	100.1	100.0	0.3
	164.0			6.0			96.9		
	132.0			1.0			100.1		
	189.0			0.0			100.7		
	169.0			0.0			100.7		
	168.0			2.0			99.4		
				0.0			100.7		
				1.0			100.1		
				0.0			100.7		
				0.0			100.7		
				2.0			99.4		
				0.0			100.7		

Cycle 10

Particle Size (uM)	Control: Particle #	Control: Average	Control: Standrad error	KD: Particle#	KD: Average	KD: Standrad error	KD: FE (Norm. to cycle 0)	KD: Average %	KD: Standrad error
0.3	32380.0	32600.2	142.0	10114.0	13297.2	445.6	86.1	73.9	1.7
	32891.0			11184.0			82.0		
	32328.0			13255.0			74.1		
	32898.0			12255.0			77.9		
	32162.0			14259.0			70.2		
	32942.0			14002.0			71.2		
				13069.0			74.8		
				13225.0			74.2		
				13355.0			73.7		
				14485.0			69.4		
				14666.0			68.7		
				15697.0			64.7		
0.5	3217.0	3464.8	69.8	968.0	1084.0	38.3	85.4	81.4	1.3
	3387.0			1225.0			76.6		
	3488.0			966.0			85.5		
	3663.0			1025.0			83.4		
	3388.0			1222.0			76.7		
	3646.0			1001.0			84.3		
				1325.0			73.2		
				1026.0			83.4		
				1029.0			83.3		
				1258.0			75.5		
				961.0			85.6		
				1002.0			84.2		
0.7	330.0	381.3	17.5	83.0	81.8	1.4	86.9	87.3	0.4
	325.0			89.0			85.2		
	417.0			85.0			86.3		
	392.0			78.0			88.4		
	405.0			82.0			87.2		
	419.0			87.0			85.8		
				85.0			86.3		
				86.0			86.1		
				75.0			89.3		
				73.0			89.8		
				81.0			87.5		
				78.0			88.4		
1.0	1296.0	1534.2	55.8	236.0	226.5	5.7	91.1	91.8	0.4
	1459.0			245.0			90.5		
	1574.0			198.0			93.8		
	1572.0			236.0			91.1		
	1641.0			225.0			91.9		
	1663.0			211.0			92.8		
				255.0			89.8		
				239.0			90.9		
				241.0			90.7		
				198.0			93.8		
				233.0			91.3		
				201.0			93.5		
2.0	1190.0	1447.7	60.2	60.0	70.8	3.5	98.8	98.0	0.2
	1355.0			65.0			98.5		
	1538.0			50.0			99.5		
	1492.0			78.0			97.5		
	1545.0			52.0			99.4		
	1566.0			76.0			97.7		
				66.0			98.4		
				76.0			97.7		
				77.0			97.6		
				87.0			96.9		
				80.0			97.4		
				83.0			97.2		
5.0	180.0	231.7	12.4	5.0	2.3	0.5	98.5	99.7	0.2
	222.0			4.0			99.0		
	245.0			5.0			98.5		
	249.0			1.0			100.3		
	226.0			1.0			100.3		
	268.0			2.0			99.8		
				2.0			99.8		
				3.0			99.4		
				2.0			99.8		
				1.0			100.3		
				2.0			99.8		
				0.0			100.7		

Mask Type: KN95				Date: 27/08/2020						
		Cycle 0								
		Control			KD					
Particle Size (uM)	Mask#	Particle#	Average	Standrad error	Particle#	Average	Standrad error	FE (Norm. to control %)	Average%	Standrad error
	1.0	35193.0			153.0			99.5		
	2.0	34476.0			551.0			98.1		
	3.0	27402.0			228.0			99.2		
	4.0	26748.0			140.0			99.5		
	5.0	23924.0			231.0			99.2		
0.3	6.0	26215.0	28993.0	1910.4	350.0	267.1	32.0	98.8	99.1	0.1
	7.0				169.0			99.4		
	8.0				295.0			99.0		
	9.0				303.0			99.0		
	10.0				300.0			99.0		
	11.0				210.0			99.3		
	12.0				275.0			99.1		
	1.0	2250.0			4.0			99.8		
	2.0	2024.0			17.0			99.1		
	3.0	1693.0			4.0			99.8		
	4.0	1646.0			7.0			99.6		
	5.0	1514.0			15.0			99.2		
0.5	6.0	2590.0	1952.8	168.7	18.0	12.3	1.5	99.1	99.4	0.1
	7.0				10.0			99.5		
	8.0				15.0			99.2		
	9.0				17.0			99.1		
	10.0				14.0			99.3		
	11.0				11.0			99.4		
	12.0				16.0			99.2		
	1.0	133.0			1.0			99.2		
	2.0	131.0			1.0			99.2		
	3.0	91.0			0.0			100.0		
	4.0	98.0			1.0			99.2		
	5.0	108.0			0.0			100.0		
0.7	6.0	152.0	118.8	9.6	1.0	0.5	0.2	99.2	99.6	0.1
	7.0				0.0			100.0		
	8.0				1.0			99.2		
	9.0				0.0			100.0		
	10.0				0.0			100.0		
	11.0				0.0			100.0		
	12.0				1.0			99.2		
	1.0	357.0			1.0			99.7		
	2.0	343.0			2.0			99.5		
	3.0	264.0			2.0			99.5		
	4.0	328.0			1.0			99.7		
	5.0	440.0			1.0			99.7		
1.0	6.0	551.0	380.5	41.2	1.0	2.3	0.4	99.7	99.4	0.1
	7.0				4.0			98.9		
	8.0				2.0			99.5		
	9.0				4.0			98.9		
	10.0				2.0			99.5		
	11.0				3.0			99.2		
	12.0				4.0			98.9		
	1.0	223.0			0.0			100.0		
	2.0	185.0			2.0			99.4		
	3.0	173.0			1.0			99.7		
	4.0	264.0			1.0			99.7		
	5.0	492.0			3.0			99.1		
2.0	6.0	642.0	329.8	78.5	1.0	1.3	0.5	99.7	99.6	0.1
	7.0				0.0			100.0		
	8.0				3.0			99.1		
	9.0				5.0			98.5		
	10.0				0.0			100.0		
	11.0				0.0			100.0		
	12.0				0.0			100.0		
	1.0	42.0			0.0			100.0		
	2.0	48.0			0.0			100.0		
	3.0	30.0			0.0			100.0		
	4.0	65.0			0.0			100.0		
	5.0	183.0			0.0			100.0		
5.0	6.0	199.0	94.5	30.9	0.0	0.1	0.1	100.0	99.9	0.1
	7.0				0.0			100.0		
	8.0				0.0			100.0		
	9.0				1.0			98.9		
	10.0				0.0			100.0		
	11.0				0.0			100.0		
	12.0				0.0			100.0		

Mask Type: KN95

Cycle 1

Particle Size (uM)	Control Particle #	Control Average	Control Standard error	KD Particle #	KD Average	KD Standrad error	FE (Norm. to cycle 0)	Average %	Standard error
0.3	32410.0	30106.3	1279.7	322.0	339.4	43.9	99.8	99.8	0.1
	27668.0			436.0			99.4		
	26065.0			536.0			99.1		
	34687.0			392.0			99.6		
	30392.0			202.0			100.2		
	29416.0			310.0			99.9		
				579.0			99.0		
				300.0			99.9		
				87.0			100.6		
				223.0			100.2		
				496.0			99.2		
				190.0			100.3		
0.5	1978.0	2153.0	280.1	23.0	32.2	5.4	99.5	99.1	0.3
	1466.0			54.0			98.1		
	1367.0			41.0			98.7		
	3108.0			39.0			98.8		
	2701.0			20.0			99.7		
	2298.0			32.0			99.1		
				47.0			98.4		
				23.0			99.5		
				2.0			100.5		
				20.0			99.7		
				69.0			97.4		
				16.0			99.9		
0.7	120.0	102.2	5.0	1.0	0.8	0.4	99.4	99.6	0.4
	83.0			4.0			96.5		
	101.0			1.0			99.4		
	106.0			0.0			100.4		
	106.0			0.0			100.4		
	97.0			0.0			100.4		
				4.0			96.5		
				0.0			100.4		
				0.0			100.4		
				0.0			100.4		
				0.0			100.4		
				0.0			100.4		
1.0	406.0	324.3	18.7	0.0	2.8	0.6	100.6	99.7	0.2
	281.0			3.0			99.7		
	288.0			5.0			99.1		
	337.0			4.0			99.4		
	330.0			0.0			100.6		
	304.0			1.0			100.3		
				8.0			98.1		
				3.0			99.7		
				2.0			100.0		
				4.0			99.4		
				2.0			100.0		
				2.0			100.0		
2.0	420.0	320.7	23.1	1.0	2.1	0.5	100.1	99.7	0.2
	251.0			1.0			100.1		
	338.0			5.0			98.8		
	301.0			2.0			99.8		
	316.0			0.0			100.4		
	298.0			0.0			100.4		
				3.0			99.5		
				5.0			98.8		
				1.0			100.1		
				4.0			99.1		
				3.0			99.5		
				0.0			100.4		
5.0	127.0	91.3	7.8	0.0	0.8	0.4	100.1	99.3	0.4
	69.0			0.0			100.1		
	91.0			1.0			99.0		
	88.0			0.0			100.1		
	84.0			1.0			99.0		
	89.0			0.0			100.1		
				5.0			94.6		
				1.0			99.0		
				0.0			100.1		
				0.0			100.1		
				1.0			99.0		
				0.0			100.1		

Cycle 2

Particle Size (uM)	Control Particle #	Control Average	Control Standard error	KD Particle #	KD Average	KD Standrad error	FE (Norm. to cycle 0)	Average%	Standard error
0.3	20065.0	15862.9	492.3	19.0	26.6	4.6	100.8	100.7	0.0
	16317.0			26.0			100.7		
	15983.0			21.0			100.8		
	15327.0			35.0			100.7		
	15753.0			46.0			100.6		
	15267.0			65.0			100.5		
	15035.0			14.0			100.8		
	14731.0			10.0			100.8		
	14890.0			21.0			100.8		
	15261.0			31.0			100.7		
				15.0			100.8		
				16.0			100.8		
0.5	2454.0	1918.1	65.7	4.0	4.5	1.4	100.4	100.4	0.1
	1836.0			0.0			100.6		
	2015.0			3.0			100.4		
	1932.0			4.0			100.4		
	1909.0			11.0			100.0		
	1905.0			11.0			100.0		
	1845.0			1.0			100.6		
	1716.0			2.0			100.5		
	1749.0			14.0			99.9		
	1820.0			3.0			100.4		
				0.0			100.6		
				1.0			100.6		
0.7	201.0	168.4	5.1	0.0	0.2	0.1	100.4	100.3	0.1
	172.0			1.0			99.8		
	175.0			0.0			100.4		
	164.0			1.0			99.8		
	186.0			0.0			100.4		
	161.0			0.0			100.4		
	154.0			0.0			100.4		
	144.0			0.0			100.4		
	164.0			0.0			100.4		
	163.0			0.0			100.4		
				0.0			100.4		
				0.0			100.4		
1.0	605.0	531.9	15.9	0.0	0.3	0.1	100.6	100.6	0.0
	511.0			0.0			100.6		
	618.0			1.0			100.4		
	529.0			0.0			100.6		
	568.0			0.0			100.6		
	473.0			1.0			100.4		
	528.0			0.0			100.6		
	481.0			0.0			100.6		
	486.0			1.0			100.4		
	520.0			0.0			100.6		
				0.0			100.6		
				0.0			100.6		
2.0	333.0	371.9	18.9	1.0	0.3	0.2	100.1	100.3	0.1
	407.0			0.0			100.4		
	473.0			0.0			100.4		
	399.0			0.0			100.4		
	442.0			1.0			100.1		
	399.0			0.0			100.4		
	325.0			0.0			100.4		
	326.0			0.0			100.4		
	312.0			2.0			99.9		
	303.0			0.0			100.4		
				0.0			100.4		
				0.0			100.4		
5.0	38.0	68.7	5.9	0.0	0.1	0.1	100.1	100.0	0.1
	73.0			0.0			100.1		
	106.0			0.0			100.1		
	88.0			0.0			100.1		
	69.0			0.0			100.1		
	73.0			0.0			100.1		
	59.0			0.0			100.1		
	59.0			0.0			100.1		
	55.0			1.0			98.6		
	67.0			0.0			100.1		
				0.0			100.1		
				0.0			100.1		

Mask Type: KN95	Cycle 3									Cycle 4								
	Control			KD						Control			KD					
Particle Size (uM)	Particle #	Average	Standrad error	Particle #	Average	Standrad error	FE (Norm. to cycle 0)	Average %	Standrad error	Particle #	Average	Standrad error	Particle #	Average	Standrad error	FE (Norm. to cycle 0)	Average %	Standrad error
0.3	14207.0	13726.3	199.0	38.0	25.6	5.0	100.6	100.7	0.0	24952.0	24188.3	1478.1	36.0	57.3	10.6	100.8	100.7	0.0
	13596.0			4.0			100.9			25467.0			123.0			100.4		
	13012.0			17.0			100.8			25707.0			110.0			100.4		
	13852.0			57.0			100.5			25545.0			97.0			100.5		
	13483.0			4.0			100.9			24468.0			21.0			100.8		
	13084.0			8.0			100.8			26145.0			31.0			100.8		
	13685.0			15.0			100.8			27187.0			26.0			100.8		
	13367.0			25.0			100.7			14035.0			20.0			100.8		
	13773.0			44.0			100.6						41.0			100.7		
	15204.0			45.0			100.6						38.0			100.7		
				23.0			100.7						64.0			100.6		
				27.0			100.7						81.0			100.6		
0.5	1591.0	1491.7	45.4	2.0	2.8	0.8	100.5	100.4	0.1	1475.0	1651.4	158.3	1.0	4.2	1.0	100.5	100.3	0.1
	1543.0			0.0			100.6			1505.0			7.0			100.2		
	1419.0			0.0			100.6			1507.0			9.0			100.1		
	1501.0			7.0			100.1			1397.0			8.0			100.1		
	1426.0			0.0			100.6			1490.0			2.0			100.5		
	1372.0			3.0			100.4			1676.0			1.0			100.5		
	1371.0			2.0			100.5			2740.0			3.0			100.4		
	1392.0			0.0			100.6			1421.0			0.0			100.6		
	1459.0			4.0			100.3						1.0			100.5		
	1843.0			8.0			100.1						7.0			100.2		
				2.0			100.5						3.0			100.4		
				5.0			100.3						8.0			100.1		
0.7	136.0	102.5	4.8	0.0	0.3	0.3	100.4	100.1	0.3	100.0	101.1	8.0	0.0	0.2	0.1	100.4	100.2	0.1
	94.0			1.0			99.4			98.0			0.0			100.4		
	98.0			0.0			100.4			110.0			0.0			100.4		
	111.0			0.0			100.4			96.0			1.0			99.4		
	93.0			0.0			100.4			88.0			0.0			100.4		
	96.0			0.0			100.4			86.0			1.0			99.4		
	83.0			0.0			100.4			152.0			0.0			100.4		
	92.0			0.0			100.4			79.0			0.0			100.4		
	107.0			0.0			100.4						0.0			100.4		
	115.0			0.0			100.4						0.0			100.4		
				3.0			97.5						0.0			100.4		
				0.0			100.4						0.0			100.4		
1.0	439.0	370.1	11.4	1.0	0.3	0.2	100.3	100.5	0.1	351.0	330.6	11.5	0.0	0.3	0.2	100.6	100.5	0.1
	364.0			0.0			100.6			353.0			0.0			100.6		
	361.0			0.0			100.6			366.0			2.0			100.0		
	387.0			0.0			100.6			319.0			1.0			100.3		
	418.0			0.0			100.6			355.0			0.0			100.6		
	316.0			0.0			100.6			325.0			0.0			100.6		
	337.0			1.0			100.3			308.0			0.0			100.6		
	359.0			0.0			100.6			268.0			0.0			100.6		
	359.0			2.0			100.1						0.0			100.6		
	361.0			0.0			100.6						1.0			100.3		
				0.0			100.6						0.0			100.6		
				0.0			100.6						0.0			100.6		
2.0	494.0	376.1	17.1	0.0	0.2	0.1	100.4	100.4	0.0	468.0	373.5	30.3	0.0	0.3	0.2	100.4	100.3	0.1
	422.0			0.0			100.4			461.0			0.0			100.4		
	354.0			0.0			100.4			437.0			1.0			100.1		
	352.0			0.0			100.4			341.0			2.0			99.9		
	383.0			0.0			100.4			397.0			0.0			100.4		
	327.0			0.0			100.4			376.0			0.0			100.4		
	305.0			0.0			100.4			235.0			0.0			100.4		
	344.0			0.0			100.4			273.0			0.0			100.4		
	399.0			1.0			100.1						1.0			100.1		
	381.0			0.0			100.4						0.0			100.4		
				0.0			100.4						0.0			100.4		
				1.0			100.1						0.0			100.4		
5.0	115.0	108.9	4.8	0.0	0.2	0.1	100.1	99.9	0.1	124.0	107.9	10.1	0.0	0.1	0.1	100.1	100.0	0.1
	131.0			0.0			100.1			130.0			0.0			100.1		
	102.0			0.0			100.1			117.0			0.0			100.1		
	129.0			1.0			99.2			111.0			0.0			100.1		
	121.0			0.0			100.1			125.0			0.0			100.1		
	95.0			0.0			100.1			124.0			0.0			100.1		
	95.0			1.0			99.2			46.0			0.0			100.1		
	87.0			0.0			100.1			86.0			0.0			100.1		
	112.0			0.0			100.1						1.0			99.2		
	102.0			0.0			100.1						0.0			100.1		
				0.0			100.1						0.0			100.1		
				0.0			100.1						0.0			100.1		

Mask Type: KN95

Cycle 5

	Control			KD					
Particle Size (uM)	Particle #	Average	Standrad error	Particle #	Average	Standrad error	FE (Norm. to cycle 0)	Average %	Standrad error
	16031.0			12.0			100.8		
	15957.0			42.0			100.6		
	15718.0			33.0			100.7		
	15446.0			28.0			100.7		
	15570.0			30.0			100.7		
	15555.0			39.0			100.7		
0.3		15712.8	96.2	39.0	32.0	2.9	100.7	100.7	0.0
				30.0			100.7		
				37.0			100.7		
				31.0			100.7		
				16.0			100.8		
				47.0			100.6		
	1181.0			0.0			100.6		
	1170.0			1.0			100.5		
	1167.0			0.0			100.6		
	1227.0			2.0			100.4		
	1327.0			1.0			100.5		
	1192.0			1.0			100.5		
0.5		1210.7	24.9	1.0	0.9	0.2	100.5	100.5	0.0
				1.0			100.5		
				0.0			100.6		
				1.0			100.5		
				2.0			100.4		
				1.0			100.5		
	83.0			0.0			100.4		
	84.0			0.0			100.4		
	86.0			0.0			100.4		
	100.0			1.0			99.3		
	116.0			1.0			99.3		
	96.0			0.0			100.4		
0.7		94.2	5.2	0.0	0.3	0.1	100.4	100.0	0.2
				1.0			99.3		
				0.0			100.4		
				1.0			99.3		
				0.0			100.4		
				0.0			100.4		
	313.0			0.0			100.6		
	313.0			0.0			100.6		
	321.0			0.0			100.6		
	297.0			1.0			100.3		
	364.0			0.0			100.6		
	387.0			0.0			100.6		
1.0		332.5	14.3	1.0	0.3	0.1	100.3	100.5	0.0
				1.0			100.3		
				0.0			100.6		
				0.0			100.6		
				0.0			100.6		
				0.0			100.6		
	256.0			0.0			100.4		
	224.0			0.0			100.4		
	260.0			0.0			100.4		
	235.0			1.0			100.0		
	296.0			0.0			100.4		
	330.0			0.0			100.4		
2.0		266.8	16.2	0.0	0.2	0.1	100.4	100.3	0.0
				0.0			100.4		
				0.0			100.4		
				1.0			100.0		
				0.0			100.4		
				0.0			100.4		
	47.0			0.0			100.1		
	48.0			0.0			100.1		
	36.0			0.0			100.1		
	63.0			0.0			100.1		
	61.0			0.0			100.1		
	62.0			0.0			100.1		
5.0		52.8	4.5	0.0	0.1	0.1	100.1	99.9	0.2
				0.0			100.1		
				0.0			100.1		
				1.0			98.2		
				0.0			100.1		
				0.0			100.1		

Cycle 6

	Control			KD					
Particle Size (uM)	Particle #	Average	Standrad error	Particle #	Average	Standrad error	FE (Norm. to cycle 0)	Average %	Standrad error
	10484.0			14.0			100.8		
	10891.0			16.0			100.8		
	11238.0			13.0			100.8		
	10693.0			28.0			100.6		
	10566.0			5.0			100.9		
	10113.0			1.0			100.9		
0.3		10664.2	155.7	4.0	20.0	6.0	100.9	100.7	0.1
				9.0			100.8		
				6.0			100.9		
				61.0			100.3		
				21.0			100.7		
				62.0			100.3		
	1374.0			3.0			100.4		
	1493.0			0.0			100.6		
	1401.0			1.0			100.5		
	1357.0			3.0			100.4		
	1345.0			0.0			100.6		
	1187.0			0.0			100.6		
0.5		1359.5	40.7	0.0	3.0	1.3	100.6	100.4	0.1
				3.0			100.4		
				1.0			100.5		
				10.0			99.9		
				1.0			100.5		
				14.0			99.6		
	104.0			0.0			100.4		
	99.0			0.0			100.4		
	112.0			0.0			100.4		
	110.0			1.0			99.4		
	91.0			0.0			100.4		
	101.0			0.0			100.4		
0.7		102.8	3.1	0.0	0.3	0.3	100.4	100.1	0.3
				0.0			100.4		
				0.0			100.4		
				3.0			97.5		
				0.0			100.4		
				0.0			100.4		
	338.0			0.0			100.6		
	367.0			1.0			100.3		
	327.0			0.0			100.6		
	316.0			1.0			100.3		
	295.0			0.0			100.6		
	311.0			0.0			100.6		
1.0		325.7	10.2	0.0	0.3	0.1	100.6	100.5	0.0
				1.0			100.3		
				0.0			100.6		
				0.0			100.6		
				0.0			100.6		
				0.0			100.6		
	200.0			0.0			100.4		
	244.0			0.0			100.4		
	212.0			0.0			100.4		
	201.0			1.0			99.9		
	196.0			0.0			100.4		
	154.0			0.0			100.4		
2.0		201.2	11.8	0.0	0.3	0.1	100.4	100.3	0.1
				1.0			99.9		
				0.0			100.4		
				0.0			100.4		
				1.0			99.9		
				0.0			100.4		
	25.0			0.0			100.1		
	20.0			0.0			100.1		
	24.0			0.0			100.1		
	23.0			0.0			100.1		
	29.0			0.0			100.1		
	28.0			0.0			100.1		
5.0		24.8	1.4	0.0	0.1	0.1	100.1	99.8	0.3
				0.0			100.1		
				0.0			100.1		
				0.0			100.1		
				1.0			96.1		
				0.0			100.1		

Mask Type: KN95	Cycle 7									Cycle 8								
	Control			KD						Control			KD					
Particle Size (uM)	Particle #	Average	Standra d error	Particle #	Average	Standrad error	FE (Norm. to cycle 0)	Average %	Standra d error	Particle #	Average	Standra d error	Particle #	Average	Standrad error	FE (Norm. to cycle 0)	Average %	Standra d error
0.3	21301.0	22187.8	198.0	**28.0**	**20.6**	**3.5**	**100.8**	**100.8**	**0.0**	45828.0	45457.2	285.5	**71.0**	**30.8**	**6.0**	**100.8**	**100.8**	**0.0**
	22082.0			**36.0**			**100.7**			45720.0			**37.0**			**100.8**		
	22616.0			**42.0**			**100.7**			45812.0			**13.0**			**100.9**		
	22503.0			**12.0**			**100.9**			45329.0			**32.0**			**100.8**		
	22493.0			**1.0**			**100.9**			45956.0			**35.0**			**100.8**		
	22132.0			**11.0**			**100.9**			44098.0			**42.0**			**100.8**		
				**33.0**			**100.8**						**33.0**			**100.8**		
				**10.0**			**100.9**						**61.0**			**100.8**		
				**18.0**			**100.8**						**11.0**			**100.9**		
				**19.0**			**100.8**						**23.0**			**100.9**		
				**22.0**			**100.8**						**4.0**			**100.9**		
				**15.0**			**100.8**						**7.0**			**100.9**		
0.5	1677.0	1646.2	18.5	**0.0**	**0.9**	**0.4**	**100.6**	**100.5**	**0.0**	3603.0	3777.2	78.6	**6.0**	**2.2**	**0.7**	**100.4**	**100.5**	**0.0**
	1567.0			**1.0**			**100.5**			3513.0			**3.0**			**100.5**		
	1663.0			**1.0**			**100.5**			3899.0			**0.0**			**100.6**		
	1694.0			**0.0**			**100.6**			3981.0			**1.0**			**100.6**		
	1652.0			**0.0**			**100.6**			3940.0			**0.0**			**100.6**		
	1624.0			**0.0**			**100.6**			3727.0			**4.0**			**100.5**		
				**4.0**			**100.4**						**0.0**			**100.6**		
				**3.0**			**100.4**						**1.0**			**100.6**		
				**1.0**			**100.5**						**0.0**			**100.6**		
				**0.0**			**100.6**						**0.0**			**100.6**		
				**1.0**			**100.5**						**5.0**			**100.5**		
				**0.0**			**100.6**						**6.0**			**100.4**		
0.7	**104.0**	120.3	6.4	**0.0**	**0.1**	**0.1**	**100.4**	**100.3**	**0.1**	**183.0**	187.5	13.9	**0.0**	**0.3**	**0.2**	**100.4**	**100.2**	**0.1**
	101.0			**0.0**			**100.4**			160.0			**0.0**			**100.4**		
	126.0			**0.0**			**100.4**			236.0			**2.0**			**99.3**		
	143.0			**0.0**			**100.4**			223.0			**0.0**			**100.4**		
	124.0			**0.0**			**100.4**			166.0			**0.0**			**100.4**		
	124.0			**0.0**			**100.4**			157.0			**0.0**			**100.4**		
				**1.0**			**99.6**						**0.0**			**100.4**		
				**0.0**			**100.4**						**0.0**			**100.4**		
				**0.0**			**100.4**						**0.0**			**100.4**		
				**0.0**			**100.4**						**2.0**			**99.3**		
				**0.0**			**100.4**						**0.0**			**100.4**		
				**0.0**			**100.4**						**0.0**			**100.4**		
1.0	**380.0**	386.2	4.5	**0.0**	**0.3**	**0.2**	**100.6**	**100.5**	**0.0**	**482.0**	514.8	15.1	**0.0**	**0.4**	**0.3**	**100.6**	**100.5**	**0.1**
	385.0			**1.0**			**100.3**			488.0			**1.0**			**100.4**		
	397.0			**2.0**			**100.1**			575.0			**0.0**			**100.6**		
	390.0			**0.0**			**100.6**			534.0			**0.0**			**100.6**		
	397.0			**0.0**			**100.6**			526.0			**0.0**			**100.6**		
	368.0			**0.0**			**100.6**			484.0			**0.0**			**100.6**		
				**0.0**			**100.6**						**0.0**			**100.6**		
				**0.0**			**100.6**						**0.0**			**100.6**		
				**0.0**			**100.6**						**1.0**			**100.4**		
				**0.0**			**100.6**						**3.0**			**100.0**		
				**0.0**			**100.6**						**0.0**			**100.6**		
				**0.0**			**100.6**						**0.0**			**100.6**		
2.0	**243.0**	270.0	9.4	**0.0**	**0.1**	**0.1**	**100.4**	**100.4**	**0.0**	**465.0**	458.7	22.9	**0.0**	**0.2**	**0.1**	**100.4**	**100.4**	**0.0**
	278.0			**1.0**			**100.0**			524.0			**0.0**			**100.4**		
	309.0			**0.0**			**100.4**			494.0			**0.0**			**100.4**		
	274.0			**0.0**			**100.4**			473.0			**1.0**			**100.2**		
	255.0			**0.0**			**100.4**			434.0			**1.0**			**100.2**		
	261.0			**0.0**			**100.4**			362.0			**0.0**			**100.4**		
				**0.0**			**100.4**						**0.0**			**100.4**		
				**0.0**			**100.4**						**0.0**			**100.4**		
				**0.0**			**100.4**						**0.0**			**100.4**		
				**0.0**			**100.4**						**0.0**			**100.4**		
				**0.0**			**100.4**						**0.0**			**100.4**		
				**0.0**			**100.4**						**0.0**			**100.4**		
5.0	**25.0**	30.8	2.7	**0.0**	**0.0**	**0.0**	**100.1**	**100.1**	**0.0**	**158.0**	139.7	12.6	**0.0**	**0.1**	**0.1**	**100.1**	**100.0**	**0.1**
	33.0			**0.0**			**100.1**			176.0			**0.0**			**100.1**		
	35.0			**0.0**			**100.1**			153.0			**0.0**			**100.1**		
	41.0			**0.0**			**100.1**			143.0			**0.0**			**100.1**		
	25.0			**0.0**			**100.1**			118.0			**0.0**			**100.1**		
	26.0			**0.0**			**100.1**			90.0			**0.0**			**100.1**		
				**0.0**			**100.1**						**0.0**			**100.1**		
				**0.0**			**100.1**						**0.0**			**100.1**		
				**0.0**			**100.1**						**1.0**			**99.4**		
				**0.0**			**100.1**						**0.0**			**100.1**		
				**0.0**			**100.1**						**0.0**			**100.1**		
				**0.0**			**100.1**						**0.0**			**100.1**		

Mask Type: KN95

Cycle 9

Particle Size (uM)	Control Particle #	Control Average	Control Standrad error	KD Particle #	KD Average	KD Standrad error	FE (Norm. to cycle 0)	Average %	Standrad error
0.3	57832.0	57145.7	615.4	18.0	14.5	1.0	100.9	100.9	0.0
	59334.0			20.0			100.9		
	58159.0			13.0			100.9		
	56001.0			12.0			100.9		
	55797.0			13.0			100.9		
	55751.0			17.0			100.9		
				13.0			100.9		
				13.0			100.9		
				11.0			100.9		
				9.0			100.9		
				20.0			100.9		
				15.0			100.9		
0.5	4491.0	4439.3	84.4	0.0	0.3	0.2	100.6	100.6	0.0
	4604.0			0.0			100.6		
	4689.0			0.0			100.6		
	4458.0			1.0			100.6		
	4240.0			1.0			100.6		
	4154.0			0.0			100.6		
				0.0			100.6		
				0.0			100.6		
				0.0			100.6		
				0.0			100.6		
				0.0			100.6		
				2.0			100.6		
0.7	164.0	162.0	4.7	0.0	0.3	0.2	100.4	100.2	0.1
	145.0			0.0			100.4		
	168.0			2.0			99.2		
	166.0			0.0			100.4		
	177.0			0.0			100.4		
	152.0			0.0			100.4		
				0.0			100.4		
				0.0			100.4		
				0.0			100.4		
				0.0			100.4		
				0.0			100.4		
				1.0			99.8		
1.0	581.0	672.5	23.3	0.0	0.2	0.1	100.6	100.6	0.0
	639.0			0.0			100.6		
	668.0			0.0			100.6		
	697.0			0.0			100.6		
	707.0			0.0			100.6		
	743.0			1.0			100.5		
				0.0			100.6		
				0.0			100.6		
				0.0			100.6		
				1.0			100.5		
				0.0			100.6		
				0.0			100.6		
2.0	613.0	698.8	18.3	0.0	0.1	0.1	100.4	100.4	0.0
	689.0			0.0			100.4		
	716.0			1.0			100.3		
	733.0			0.0			100.4		
	729.0			0.0			100.4		
	713.0			0.0			100.4		
				0.0			100.4		
				0.0			100.4		
				0.0			100.4		
				0.0			100.4		
				0.0			100.4		
				0.0			100.4		
5.0	120.0	157.0	10.5	0.0	0.3	0.2	100.1	99.9	0.1
	164.0			0.0			100.1		
	132.0			2.0			98.8		
	189.0			0.0			100.1		
	169.0			0.0			100.1		
	168.0			0.0			100.1		
				0.0			100.1		
				0.0			100.1		
				0.0			100.1		
				0.0			100.1		
				0.0			100.1		
				1.0			99.5		

Cycle 10

Particle Size (uM)	Control Particle #	Control Average	Control Standrad error	KD Particle #	KD Average	KD Standrad error	FE (Norm. to cycle 0)	Average %	Standrad error
0.3	32380.0	32600.2	142.0	26.0	33.1	1.9	100.8	100.8	0.0
	32891.0			25.0			100.8		
	32328.0			36.0			100.8		
	32898.0			34.0			100.8		
	32162.0			21.0			100.8		
	32942.0			39.0			100.8		
				39.0			100.8		
				33.0			100.8		
				42.0			100.8		
				35.0			100.8		
				39.0			100.8		
				28.0			100.8		
0.5	3217.0	3464.8	69.8	8.0	4.8	0.5	100.4	100.5	0.0
	3387.0			6.0			100.4		
	3488.0			5.0			100.5		
	3663.0			4.0			100.5		
	3388.0			5.0			100.5		
	3646.0			2.0			100.5		
				7.0			100.4		
				7.0			100.4		
				5.0			100.5		
				3.0			100.5		
				3.0			100.5		
				3.0			100.5		
0.7	330.0	381.3	17.5	2.0	0.6	0.3	99.9	100.2	0.1
	325.0			0.0			100.4		
	417.0			0.0			100.4		
	392.0			0.0			100.4		
	405.0			0.0			100.4		
	419.0			0.0			100.4		
				1.0			100.1		
				1.0			100.1		
				0.0			100.4		
				0.0			100.4		
				3.0			99.6		
				0.0			100.4		
1.0	1296.0	1534.2	55.8	2.0	1.1	0.4	100.5	100.5	0.0
	1459.0			0.0			100.6		
	1574.0			0.0			100.6		
	1572.0			0.0			100.6		
	1641.0			3.0			100.4		
	1663.0			1.0			100.5		
				3.0			100.4		
				0.0			100.6		
				1.0			100.5		
				3.0			100.4		
				0.0			100.6		
				0.0			100.6		
2.0	1190.0	1447.7	60.2	1.0	0.5	0.2	100.3	100.4	0.0
	1355.0			0.0			100.4		
	1538.0			0.0			100.4		
	1492.0			0.0			100.4		
	1545.0			1.0			100.3		
	1566.0			0.0			100.4		
				0.0			100.4		
				2.0			100.3		
				0.0			100.4		
				2.0			100.3		
				0.0			100.4		
				0.0			100.4		
5.0	180.0	231.7	12.4	1.0	0.1	0.1	99.7	100.1	0.0
	222.0			0.0			100.1		
	245.0			0.0			100.1		
	249.0			0.0			100.1		
	226.0			0.0			100.1		
	268.0			0.0			100.1		
				0.0			100.1		
				0.0			100.1		
				0.0			100.1		
				0.0			100.1		
				0.0			100.1		
				0.0			100.1		

Supplementary Material 5: The raw data of experimental uncertainties analysis.
